# LncRNA FAF attenuates hypoxia/ischaemia‐induced pyroptosis via the miR‐185‐5p/PAK2 axis in cardiomyocytes

**DOI:** 10.1111/jcmm.17304

**Published:** 2022-04-03

**Authors:** Jie Gu, Jian‐Zhou Shi, Ya‐Xing Wang, Liu Liu, Si‐Bo Wang, Jia‐Teng Sun, Tian‐Kai Shan, Hao Wang, Qi‐Ming Wang, Lian‐Sheng Wang

**Affiliations:** ^1^ Department of Cardiology the First Affiliated Hospital of Nanjing Medical University Nanjing China

**Keywords:** hypoxia/ischaemia, lncRNA FAF, miR‐185‐5p, PAK2, pyroptosis

## Abstract

Pyroptosis is associated with various cardiovascular diseases. Increasing evidence suggests that long noncoding RNAs (lncRNAs) have been implicated in gene regulation, but how lncRNAs participate in the regulation of pyroptosis in the heart remains largely unknown. In this study, we aimed to explore the antipyroptotic effects of lncRNA FGF9‐associated factor (FAF) in acute myocardial infarction (AMI). The expression patterns of lncRNA FAF, miR‐185‐5p and P21 activated kinase 2 (PAK2) were detected in hypoxia/ischaemia‐induced cardiomyocytes. Hoechst 33342/PI staining, lactate dehydrogenase (LDH) release assay, immunofluorescence and Western blotting were conducted to assay cell pyroptosis. The interaction between lncRNA FAF, miR‐185‐5p and PAK2 was verified by bioinformatics analysis, small RNA sequencing luciferase reporter assay and qRT‐PCR. The expression of LncRNA FAF was downregulated in hypoxic cardiomyocytes and myocardial tissues. Overexpression of lncRNA FAF could attenuate cardiomyocyte pyroptosis, improve cell viability and reduce infarct size during the procession of AMI. Moreover, lncRNA FAF was confirmed as a sponge of miR‐185‐5p and promoted PAK2 expression in cardiomyocytes. Collectively, our findings reveal a novel lncRNA FAF/miR‐185‐5p/PAK2 axis as a crucial regulator in cardiomyocyte pyroptosis, which might be a potential therapeutic target of AMI.

## 1 INTRODUCTION

Coronary heart disease (CHD) remains a major cause of morbidity and mortality worldwide, which is responsible for nearly 1.8 million deaths in Europe, and similarly 1.7 million deaths in China annually.^1^, ^2^ Acute myocardial infarction (AMI), the worst outcome of CHD, accounts for many of these deaths.^3^ Despite there has been a dramatic improvement in the overall mortality rate of AMI due to the advances in pharmacological therapy and reperfusion strategies over the past two decades,^4^ quite a number of survivors eventually suffer from chronic heart failure, a long‐term condition which leads to frequent hospitalizations, low quality of life and huge economic burden.^5^, ^6^ AMI mainly results from sudden and sustained occlusion of the coronary artery and leads to the deprivation of oxygen and nutrients within the ischaemic zone.^7^ As a result, various types of cell death, such as necrosis, apoptosis, autophagy and pyroptosis, can occur in infarcted myocardium, respectively.^8^ Among them, pyroptosis, a highly inflammatory form of programmed cell death, is characterized by cell lysis and the release of inflammatory cytokines.^9^ Pyroptosis is initially discovered as an innate immune response to certain pathogen infection in a caspase‐1‐dependent manner.^10^ Inflammasomes, molecular platforms that function as cytosolic sensors, recognize pathogen‐associated molecular patterns (PAMPs) as well as danger‐associated molecular patterns (DAMPs) and further assemble themselves into inflammasome complexes to trigger pyroptosis via the cleavage of caspase‐1. Subsequently, activated caspase‐1 hydrolyses gasdermin D (GSDMD) to its N‐terminal fragment and converts pro‐inflammatory cytokine (pro‐IL‐18 or pro‐IL‐1β) to its mature form. Once cleaved, the N‐terminus of GSDMD functions as a pore‐forming protein thus promoting cell swelling, inflammatory cytokine release, membrane rupture and ultimately pyroptotic cell death.^11^, ^12^ NACHT, LRR and PYD domains‐containing protein 3 (NLRP3), one of the most studied inflammasomes in the heart, senses multiple host‐derived danger signals, triggers sterile inflammatory responses and induces pyroptotic cell death during AMI. Excessive activation of pyroptosis contributes to the continual loss of cardiomyocytes, increased infarct size, adverse cardiac remodelling and ventricular aneurysm formation following AMI.^13^, ^14^, ^15^, ^16^ Therefore, therapies that target the pyroptosis pathway might be valuable strategies for acute myocardial infarction.

Long noncoding RNAs (LncRNAs), over 200 nucleotides in length, generally represent a class of RNA transcripts without protein‐coding capability. LncRNAs were first described as transcriptional noise and polymerase II‐transcribed byproducts with no biological function.^17^ LncRNAs can regulate gene expression by interacting with mRNAs, microRNAs (miRNAs) and RNA‐binding proteins.^18^, ^19^ Numerous studies have revealed that lncRNAs participate in multiple cardiovascular diseases (CVDs). For instance, our prior study has found that lncRNA Kcan2‐AS promotes ventricular arrhythmias by downregulating Kcan2 expression during heart failure.^20^ LncRNA CPR has been reported to be involved in cardiomyocyte proliferation and cardiac repair after myocardial injury.^21^ LncRNA FGF9‐associated factor (FAF), a recently discovered lncRNA, is downregulated in infarcted myocardium and inhibits cardiomyocyte apoptosis and cardiac fibrosis through transcriptional regulation of FGF9.^22^, ^23^ Nonetheless, the relationship between lncRNA FAF and cardiomyocyte pyroptosis is still unclear.

miRNAs are involved in various pathologic events including CVDs.^24^ Several miRNAs have been reported to modulate pyroptosis directly or indirectly in CVDs, such as miR‐223, miR‐1, miR‐30d and miR‐214.^25^ MiR‐185‐5p was reported to function as a tumour suppressor by inhibiting proliferation and inducing apoptosis in cancers.^26^, ^27^, ^28^ Recently, one study has revealed that downregulation of miR‐185‐5p expression contributes to neovascularization in endothelial cells, which further mitigates the decline in cardiac function in postinfarction mice.^29^ Previous studies have suggested that lncRNAs could bind to miRNAs and act as competitive endogenous RNAs (ceRNAs) to regulate cell biological processes, which play a vital role in myocardial infarction.^30^, ^31^ However, the exact regulatory mechanism of miR‐185‐5p remains poorly understood in cardiomyocytes. In the present study, we investigated the potential of lncRNA FAF against cardiomyocyte pyroptosis and AMI.

## 2 MATERIALS AND METHODS

### 2.1 Cell isolation, culture and establishment of hypoxia/ischaemia

Neonatal rat cardiomyocytes (NRCM) were isolated from 1‐ to 3‐day‐old Sprague‐Dawley rats through enzyme digestion as previously described.^32^ After isolation, NRCM were transferred to a cell incubator and cultured in DMEM supplemented with 10% HS, 5% FBS and 1% penicillin‐streptomycin for 24 hours. For the establishment of hypoxia/ischaemia models in vitro, NRCM were incubated with the hypoxic solution as previously described^16^ and then transferred to a hypoxia chamber. Hypoxic stimulation was maintained at 37°C with 5% CO_2_ and 1% O_2_. After 8 hours, the cells were collected for further experiments.

### 2.2 Cell transfection

FAF‐overexpressing adenovirus vector (Adv‐FAF), adenovirus containing FAF siRNA (5'‐GCAGUUUCUCUUUCAUUCUTT‐3’) and corresponding control vectors with the enhanced green fluorescent protein (EGFP) were synthesized by GeneChem (Shanghai, China). The PAK2 inhibitor FRAX597 (C29H28ClN7OS; M.W. 558.10) was synthesized by Selleckchem (Houston, USA) and used at 5 µM for 12 hours. PAK2 expression plasmid and control vector were synthesized by GeneChem (Shanghai, China). miR‐185‐5p mimic (5'‐UGGAGAGAAAGGCAGUUCCUGA‐3’) and inhibitor (5'‐UCAGGAACUGCCUUUCUCUCCA‐3’) were acquired from RiboBio (Guangzhou, China) to alter miR‐185‐5p expression in vitro. NRCM were transfected with Adv‐FAF, si‐FAF, OE‐PAK2, miR‐185‐5p mimics and inhibitors, respectively, for 12 hours following the recommendations from the manufacturer.

### 2.3 Establishment of MI Rat model

All animal trials were approved by the ethical committees of Nanjing Medical University and conducted under the guidance of the use of laboratory animals in biomedical and behavioural experiments. (No. 85–23, US National Institutes of Health). Male Sprague‐Dawley rats were acquired from the Animal Core Facility of Nanjing Medical University.

The animal experiments were divided into two parts. In the first part of the study, rats were anaesthetized and kept ventilated through an animal ventilator. Rat models of MI were established by surgical obstruction of the left anterior descending coronary artery (LAD). For determination of the effect of lncRNA FAF overexpression on rats with MI, Adv‐FAF or Adv‐EGFP adenovirus (1 × 10^10^/100 µl) were intramyocardially injected into the ligation area after ligation of the LAD. For the sham group, rats also received similar surgical procedures but with no ligation. One week later, echocardiography was performed on all rats. Then, heart tissues were collected for further experiments.

In the second part of the study, rats were randomly divided into sham, MI + Adv‐FAF + Agomir‐NC, MI + Adv‐FAF + Agomir‐185‐5p, MI + Adv‐FAF + DMSO, and MI + Adv‐FAF + FRAX597 group. Agomir‐185‐5p (5’‐UGGAGAGAAAGGCAGUUCCUGA‐3’) and Agomir‐NC (5’‐UUUGUACUACACAAAAGUACUG‐3’) were purchased from RiboBio (Guangzhou, China). Rats received Agomir‐185‐5p or Agomir‐NC administration through intraperitoneal injection for 2 weeks (80 mg/kg/day).^33^ FRAX597, dissolved in 10% (PEG400:Tween 80:PVP‐K30 – 90:5:5) 15% Vitamin E‐TPGS and 75% of HPC (0.5%) in 50 mM citrate buffer (pH 3), was administered by oral gavage at 90 mg/kg/day for 2 weeks.^34^ After administration, MI surgery and lncRNA FAF transfection were performed on rats same as the first part. After 7 postoperative days, hearts were then harvested for TTC staining.

### 2.4 Western blot analysis

A total protein extraction kit (Keygen Biotech, Nanjing, China), which consists of lysis buffer, phosphatase inhibitor, protease inhibitor and phenylmethylsulfonyl fluoride (PMSF), was used to extract protein from cells and heart tissues. Protein samples were quantified by BCA assays (Thermo Fisher, USA). The samples were separated using 10% or 12.5% SDS‐PAGE and transferred onto PVDF membranes. Then, 5% non‐fat milk was used as a blocking buffer. The membranes were incubated with primary antibodies against tubulin (1:10,000; Bioworld; cat. no. AP0064), caspase‐1 (1:1000; Proteintech; cat. no. 22915–1‐AP), cleaved caspase‐1 (1:500; Cell Signaling Technology; cat. no.89332), IL‐18 (1:1000; Proteintech; cat. no. 10663–1‐AP), GSDMD (1:1000; Cell Signaling Technology; cat. no.93709), NLRP3 (1:1000; ABclonal; cat. no. A5652), IL‐1β (1:1000; Proteintech; cat. no. 16806–1‐AP), PAK1 (1:1000; Proteintech, cat. no. 21401–1‐AP), PAK2 (1:200; Santa Cruz Biotechnology, cat. no. sc‐373740) and caspase‐7 (1:1000; Cell Signaling Technology; cat. no. 9492) at 4℃ overnight. The immunoblots were visualized by an Enhanced Chemiluminescence Kit (Thermo Scientific, USA) and semi quantified with tubulin as the internal control.

### 2.5 Quantitative real‐time polymerase chain reaction

Total RNA was extracted from NRCM and rat heart tissues by TRIzol (Invitrogen, USA). Then, RNA was quantified and reverse transcribed into cDNA using a PrimeScript™ RT kit (TaKaRa, Japan). Quantitative real‐time polymerase chain reaction (qRT‐PCR) was carried out using SYBR Green kit (Applied Biosystems, USA) on an ABI 7900 Real‐Time PCR System (Applied Biosystems, USA). Gene expression was normalized to that of 18s, β‐actin or U6 through the 2^−ΔΔCT^ method. The specific primer sequences are shown in Table 1 and Table S1

**TABLE 1:** Specific primer list.

Target primers	Sequences
LncRNA FAF‐F	CGCTAAAGGCACAGGGTCAG
LncRNA FAF‐R	CACCAACCTTTCCCTTCCAGTC
U6‐F	CTCGCTTCGGCAGCACA
U6‐R	AACGCTTCACGAATTTGCGT
β‐actin‐F	CACCCGCGAGTACAACCTTC
β‐actin‐R	CCCATACCCACCATCACACC
miR−185‐5p‐F	CGCGTGGAGAGAAAGGCAGT
miR−185‐5p‐R	AGTGCAGGGTCCGAGGTATT
PAK2‐F	GGATGAGCAGCACCATTT
PAK2‐R	AATCAGACGGCGGAGAA

Abbreviations

F, forward and R, reverse.

### 2.6 Hoechst 3342/PI staining

The cell membrane lost its integrity upon the activation of pyroptosis.^35^ Hoechst 3342/PI double staining was performed to assess the cell membrane damage caused by pyroptosis. In brief, NRCM were cultured in 24‐well plates followed by transfection and hypoxia/ischaemia. Then, Hoechst 33342 (Sigma, USA) and PI (Sigma, USA) fluorescent dyes were mixed with culture medium. The cells were kept in the dark and stained at room temperature. Following 20 min of staining, the cells were examined under a fluorescence microscope. The percentage of PI‐positive cells was determined at ×200 magnification.

### 2.7 Immunofluorescence staining

Immunofluorescence staining was used to examine NLRP3 inflammasome activation in cardiomyocytes. Briefly, cells were fixed with 4% PFA at room temperature for 30 min. Following permeabilization with 0.5% Triton X‐100 for 20 min, cells were blocked with 10% goat serum for 1 hour at room temperature and further incubated with α‐actinin (1:150; Sigma; cat. no. A7811) and NLRP3 (1:200; ABclonal; cat. no. A5652) at 4°C overnight in the dark. Then, the cells were stained with the proper secondary antibody. DAPI (Sigma, USA) was applied to label the nucleus. The staining results were acquired via fluorescence microscopy (Carl Zeiss, Germany).

### 2.8 LDH release assay

The LDH release in the supernatants was determined by an LDH cytotoxicity assay kit (Beyotime, Shanghai, China). Cells were cultured in 96‐well plates. Subsequently, the LDH detection agent was added following the manufacturer's instructions. The absorbance of the supernatants was determined at 490 nm, and LDH release was calculated by a standard curve.

### 2.9 TTC staining

The infarct size was measured by triphenyl tetrazolium chloride (TTC) staining. Hearts were washed with normal saline and sliced into 2 mm coronary sections. Subsequently, the slices were incubated in 1% TTC at 37°C for 20 min and fixed in 10% formalin to measure the infarct area. The surviving tissue turned deep red, whereas the infarcted tissue bleached.

### 2.10 Echocardiography

For assessment of the cardiac structure and function, rats were anaesthetized and examined by a transthoracic echocardiography detection system (Visuasonics, Canada) as previously described.^23^ Left ventricular ejection fraction (LVEF) and left ventricular fractional shortening (LVFS) were automatically measured by a microcomputer of the echocardiography system.

### 2.11 Scanning electron microscopy

Neonatal rat cardiomyocytes were harvested and sent to the Electron Microscopy Centre of Nanjing Medical University for further preparations. Samples were viewed under Scanning Electron Microscopy (SEM) (JEOL JSM‐7900F SEM system).

### 2.12 Transmission electron microscopy

Fresh myocardium was cut into fragments of 1 mm sections and fixed in 4% glutaraldehyde and 1% osmic acid overnight at 4°C overnight. Then, the samples were sent to the Electron Microscopy Centre of Nanjing Medical University for further preparations. The ultrastructure of myocardium was examined by Transmission Electron Microscopy (TEM) (JEOL JEM‐1400Flash TEM system).

### 2.13 Small RNA sequencing and differential miRNA expression analysis

Total RNA was isolated from FAF‐overexpressing NRCM and negative controls, respectively. Differential miRNA expression profiles in the FAF‐overexpressing NRCM were determined by high‐throughput miRNA sequencing. Small RNA library preparation and sequencing were performed by Beijing Novogene Co., Ltd. (Beijing, China) as follows: Briefly, a total of 3 µg RNA of each sample were collected for further processing. Construction of sequencing libraries was accomplished by using an EBNext^®^Multiplex Library Prep Set (NEB, USA) following the recommendations from the manufacturer. Qubit 2.0 (Life Technologies, USA) was used to measure the library concentration. Then, an Agilent Bioanalyzer 2100 system (Agilent Technologies, CA) was used for quality control. Finally, the library preparations were sequenced on an Illumina HiSeq 2500 platform (Illumina, Inc.). RNA abundances were evaluated and normalized by the Transcript Per Million (TPM).^36^ The Benjamini and Hochberg method was utilized for P‐value adjustment. The criteria of differentially expressed miRNAs were set as the absolute fold change >1.5 and adjusted *P*‐values <0.05.

### 2.14 Luciferase reporter assay

Luciferase reporter assays were performed to verify the interactions among lncRNA FAF, miR‐185‐5p and PAK2. The putative binding sites of miR‐185‐5p with lncRNA FAF and miR‐185‐5p with PAK2 were determined through RNAhybrid software.^37^ Wild‐type and mutant sequences (mut‐lncRNA FAF, wt‐lncRNA FAF, mut‐PAK2 and wt‐PAK2) were designed and cloned into the pGL3/Luciferase vector (Promega, USA). HEK293T cells were incubated with the vectors mut‐lncRNA FAF, wt‐lncRNA FAF, mut‐PAK2 and wt‐PAK2, respectively, and then cotransfected with mimics (30 nM, RiboBio, Guangzhou, China) or negative controls (30 nM, RiboBio, Guangzhou, China) for 48 hours. Subsequently, cells were collected for further measurement. The luciferase activity was determined by using a Dual‐Lumi™ II Luciferase Kit (Beyotime, Shanghai, China).

### 2.15 Statistical analysis

Statistical analyses were accomplished by using SPSS 19.0 software (IBM, USA). All experimental results are displayed as mean ± SD. The two‐tailed Student's t‐test was performed to determine the difference between two groups, while the one‐way ANOVA followed by Tukey's test was applied for multiple comparisons. *p* < 0.05 indicated a significant difference. Graphs were generated by GraphPad Prism 8.0 (GraphPad Software, USA).

## 3 RESULTS

### 3.1 LncRNA FAF improved cardiac function and suppressed pyroptosis in rats with MI

**FIGURE 1:**
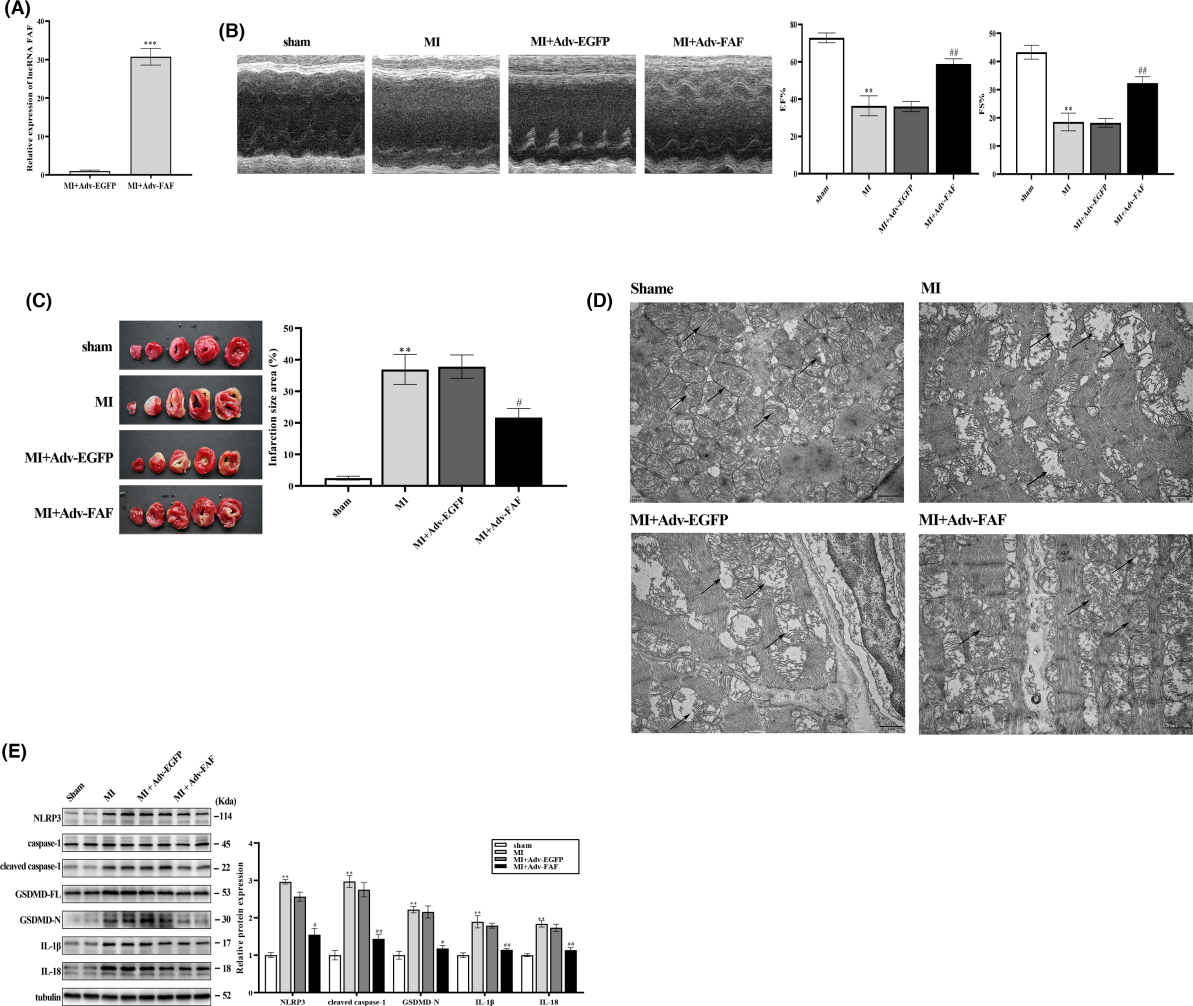
LncRNA FAF improved cardiac function and suppressed pyroptosis in rats with MI. (A)The transfection efficiency of Adv‐FAF in MI heart tissues. (B) The echocardiographic parameters LVEF and LVFS were evaluated in each group. (C) The infarct size of each group was measured by TTC staining. (D) Transmission electron microscopy analysis of myocardial injury at the ultrastructure level. The arrow points to the mitochondria of cardiomyocytes. Scale bar = 1 μm. *n *= 5 per group (E) Western blotting was performed to determine the effect of lncRNA FAF on pyroptosis‐related proteins in MI heart tissues. *n* = 5 rats per group, ^*^p < 0.05, ^**^p < 0.01, ^***^p < 0.001, *vs* the sham group; *^#^p* < 0.05, *^##^p* < 0.01 *vs* the MI +Adv‐EGFP group

Our previous study found that expression of lncRNA FAF was downregulated in the ischaemic heart and associated with cardiomyocyte apoptosis.^22^ To investigate the therapeutic efficacy of lncRNA FAF and potential antipyroptotic function in vivo, an adenovirus with FAF‐overexpressing vector was synthesized and packaged in HEK293 cells. Then, we overexpressed lncRNA FAF in rats with MI by intramyocardially injecting Adv‐FAF. After one week, qRT‐PCR was conducted to assess the overexpression efficiency of lncRNA FAF (Figure 1A). Cardiac function was evaluated by echocardiography one week after MI surgery. The echocardiographic parameters of LVFS and LVEF were increased in MI + Adv‐FAF group compared with MI + Adv‐EGFP group (Figure 1B). Consistently, TTC staining analysis showed that the infarct size in the MI + Adv‐FAF group was smaller than that in the MI and MI + Adv‐EGFP groups (Figure 1C). Transmission electron microscopy was used to assess the myocardial injury at the ultrastructure level. As shown in Figure 1D, the TEM results of myocardium in MI and MI + Adv‐EGFP group showed severe myofilaments lysis, loss of plasma membrane integrity, mitochondria swelling, cristae disappearance and other ultrastructure pathological changes in contrast to the Sham group. Most importantly, the pathology of the myocardium in the MI + Adv‐FAF group was significantly ameliorated. The disarrangement of myofilaments, plasma membrane destruction and mitochondria swelling were greatly improved compared with MI and MI + Adv‐EGFP group. Furthermore, measurement of pyroptosis‐related proteins revealed that overexpressing lncRNA FAF suppressed pyroptosis in ischaemic hearts (Figure 1E). These results indicated that lncRNA FAF could suppress myocardium pyroptosis induced by hypoxia/ischaemia and promote cardiac function recovery after MI.

### 3.2 LncRNA FAF suppressed hypoxia/ischaemia‐induced cardiomyocyte pyroptosis

**FIGURE 2:**
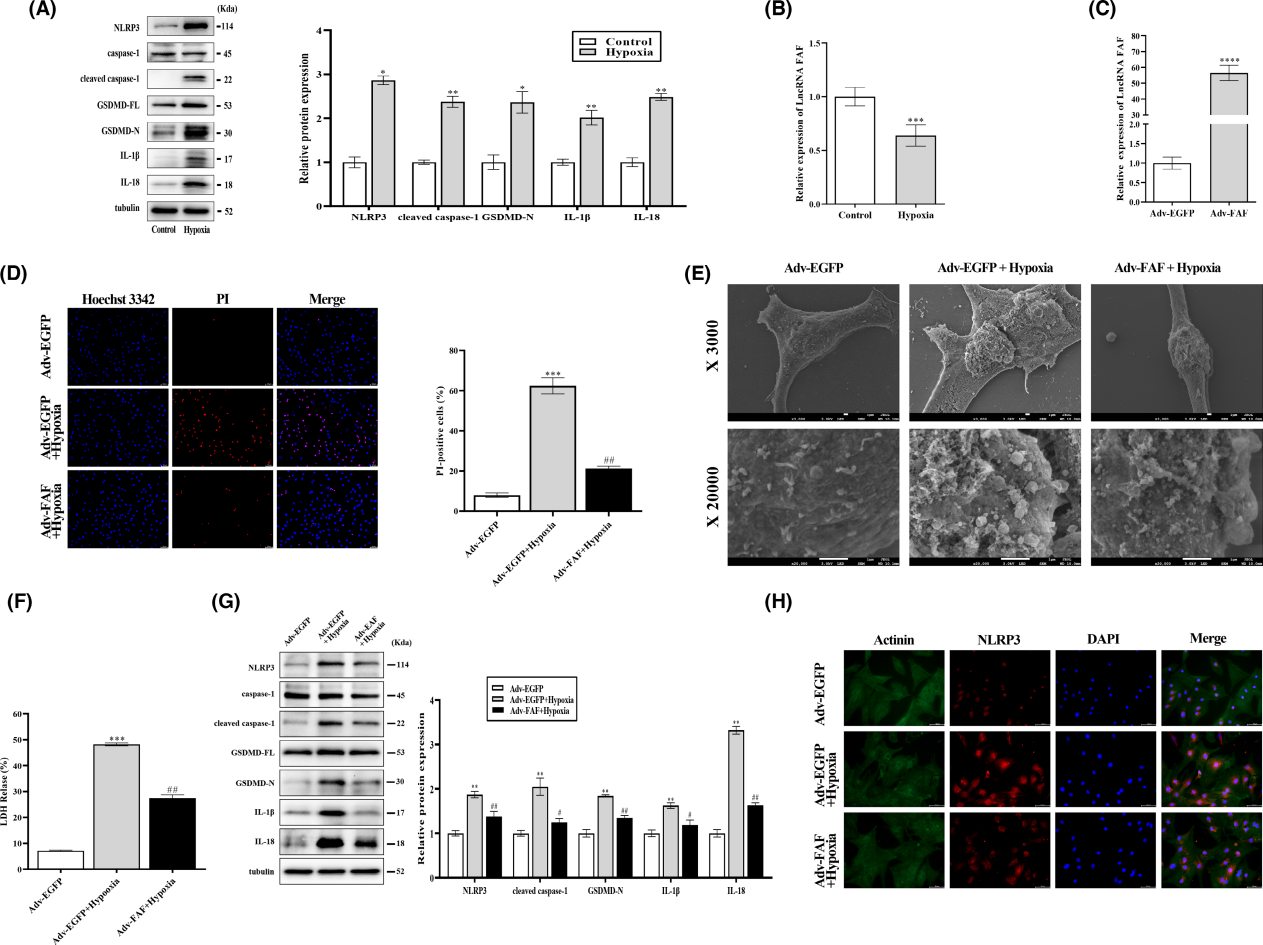
LncRNA FAF suppressed hypoxia/ischaemia‐induced cardiomyocyte pyroptosis. (A) Western blotting was performed to determine the effect of lncRNA FAF on pyroptosis‐related proteins in hypoxic NRCM. (B) The expression level of lncRNA FAF in hypoxic and normoxic cardiomyocytes was determined by qRT‐PCR. NRCM were transfected with Adv‐FAF and Adv‐EGFP, respectively, and subsequently exposed to hypoxia/ischaemia. (C) The transfection efficiency of Adv‐FAF in NRCM. (D) Pyroptosis was assessed by the proportion of PI‐positive cells in each group. Magnification: ×200. Scale bar = 50 μm. (E) Scanning electron micrographs of NRCM Magnification: ×3000. And ×20000. Scale bar = 1 μm. (F) LDH release was detected in hypoxic cardiomyocytes. (G) Western blotting was performed to determine the effect of lncRNA FAF on pyroptosis‐related proteins in hypoxic NRCM transfected with Adv‐FAF. (H) NLRP3 expression in hypoxic NRCM was determined by Immunofluorescence staining. Magnification: ×400. Scale bar = 50 μm. *n* = 3, *p < 0.05, **p < 0.01, ***p < 0.001, ****p < 0.0001 *vs* the control group or Adv‐EGFP group; *^#^p* < 0.05, ^##^ *p* < 0.01 *vs* the Adv‐EGFP +hypoxia group

To further investigate the effects of lncRNA FAF on cardiomyocyte pyroptosis, NRCM were subjected to hypoxia/ischaemia conditions for 8 hours to induce pyroptosis (Figure S1). Western blot analysis showed that the expression levels of pyroptosis‐related proteins were significantly increased in the hypoxia/ischaemia group, which indicated the activation of pyroptosis under hypoxia/ischaemia (Figure 2A). Consistent with our previous study,^22^ downregulation of lncRNA FAF was detected in the hypoxia/ischaemia injured cardiomyocytes by qRT‐PCR (Figure 2B). NRCM were further transfected with Adv‐FAF or Adv‐EGFP, following by hypoxia/ischaemia stimulus for 8 hours. qRT‐PCR results indicated that lncRNA FAF was markedly upregulated in the Adv‐FAF group (Figure 2C). The loss of membrane integrity is an important cytomorphology of pyroptosis. Hoechst 3342/PI staining showed that overexpression of lncRNA FAF in Hypoxia + Adv‐FAF group preserved the cell membrane integrity from hypoxia/ischaemia‐induced pyroptosis (Figure 2D). Next, NRCM were evaluated for cell morphology via scanning electron microscope (SEM) at the microstructure level (Figure 2E). In contrast to the normoxia group, NRCM subjected to hypoxia/ischaemia appeared more rounded, accompanied by the appearance of bulging masses and occasional blebs across the cell surface, which resemble ‘pyroptotic bodies’ reported by Chen et al..^38^ Most importantly, NRCM in the Hypoxia + Adv‐FAF group significantly reduced the abnormalities in morphology compared with Hypoxia + Adv‐EGFP group. The results of LDH release detection indicated that lncRNA FAF overexpression enhanced cell viability in the hypoxia/ischaemia injured cardiomyocytes (Figure 2F). Finally, we examined the regulatory effects of lncRNA FAF on the expression of pyroptosis‐related proteins. The protein levels of NLRP3, cleaved caspase‐1, GSDMD‐N, IL‐1β and IL‐18 decreased pronouncedly in the Adv‐FAF group compared with the Adv‐EGFP group under hypoxia/ischaemia (Figure 2G). In addition, we performed immunofluorescence staining to detect NLRP3 activity in primary cardiomyocytes. Compared with transfection of negative control adenovirus, transfection of FAF‐overexpressing adenovirus suppressed the activation of the NLRP3 inflammasome in hypoxic cardiomyocytes (Figure 2H). Collectively, these findings suggested that overexpression of lncRNA FAF could protect cardiomyocytes against cardiomyocyte pyroptosis.

### 3.3 Interfering with lncRNA FAF expression aggravated hypoxia/ischaemia‐induced pyroptosis in cardiomyocytes

**FIGURE 3:**
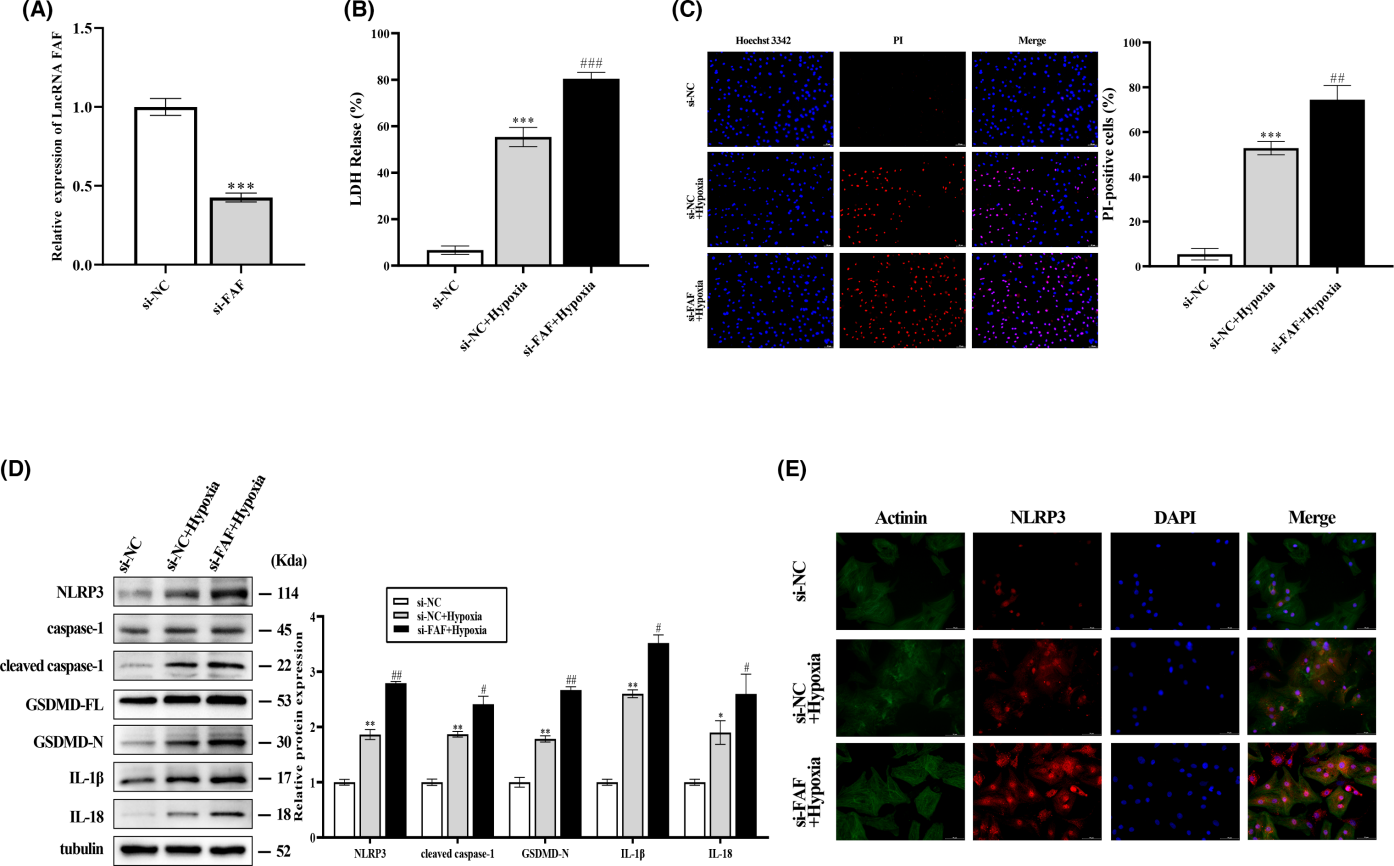
Interfering with lncRNA FAF expression aggravated hypoxia/ischaemia‐induced pyroptosis in cardiomyocytes. NRCM were transfected with si‐FAF and si‐NC, respectively, and subsequently exposed to hypoxia/ischaemia. (A) The transfection efficiency of si‐FAF was measured by qRT‐PCR. (B and C) LDH release and Hoechst 33342/PI staining were performed to assess cell pyroptosis in each group. Magnification: ×200. Scale bar  = 50 μm. (D) Western blot was performed to determine the effect of lncRNA FAF on pyroptosis‐related proteins in hypoxic NRCM transfected with si‐FAF. € NLRP3 expression was detected by Immunofluorescence staining in hypoxic cardiomyocytes. Magnification: ×400. Scale bar  = 50 μm. *n* = 3, *p < 0.05, **p < 0.01, ***p < 0.001 *vs* the si‐NC group; *^#^p* < 0.05, *^##^p* < 0.01 *vs* si‐NC +hypoxia group

Knowing that lncRNA FAF positively regulates pyroptosis in cardiomyocytes, we next examined whether downregulation of lncRNA FAF aggravated cardiomyocyte pyroptosis. NRCM were transfected with si‐FAF and incubated under hypoxia/ischaemia conditions for 8 hours. The successful transfection of si‐FAF in NRCM was verified by qRT‐PCR (Figure 3A). Knockdown of lncRNA FAF increased the release of LDH in si‐FAF + Hypoxia cells, compared with the si‐NC + hypoxia group (Figure 3B). Meanwhile, the PI‐positive rates increased in the hypoxia/ischaemia injury group, and the si‐FAF + Hypoxia group had the highest rate according to the assessment of Hoechst 3342/PI staining (Figure 3C). Additionally, Western blot analysis revealed that pyroptosis‐related protein levels were significantly increased in the hypoxia/ischaemia injured cardiomyocytes, which was further worsened by transfection of si‐FAF (Figure 3D). Immunofluorescence staining measurements showed that si‐FAF transfection promoted the activation of the NLRP3 inflammasome in hypoxia/ischaemia injured cardiomyocytes, compared with the other two groups (Figure 3E). Taken together, these results indicated that interfering with lncRNA FAF expression aggravated pyroptosis in hypoxia/ischaemia injured cardiomyocytes.

### 3.4 Expression profile of miRNAs in the FAF‐overexpressing NRCM and validation of potential target miRNAs

**FIGURE 4:**
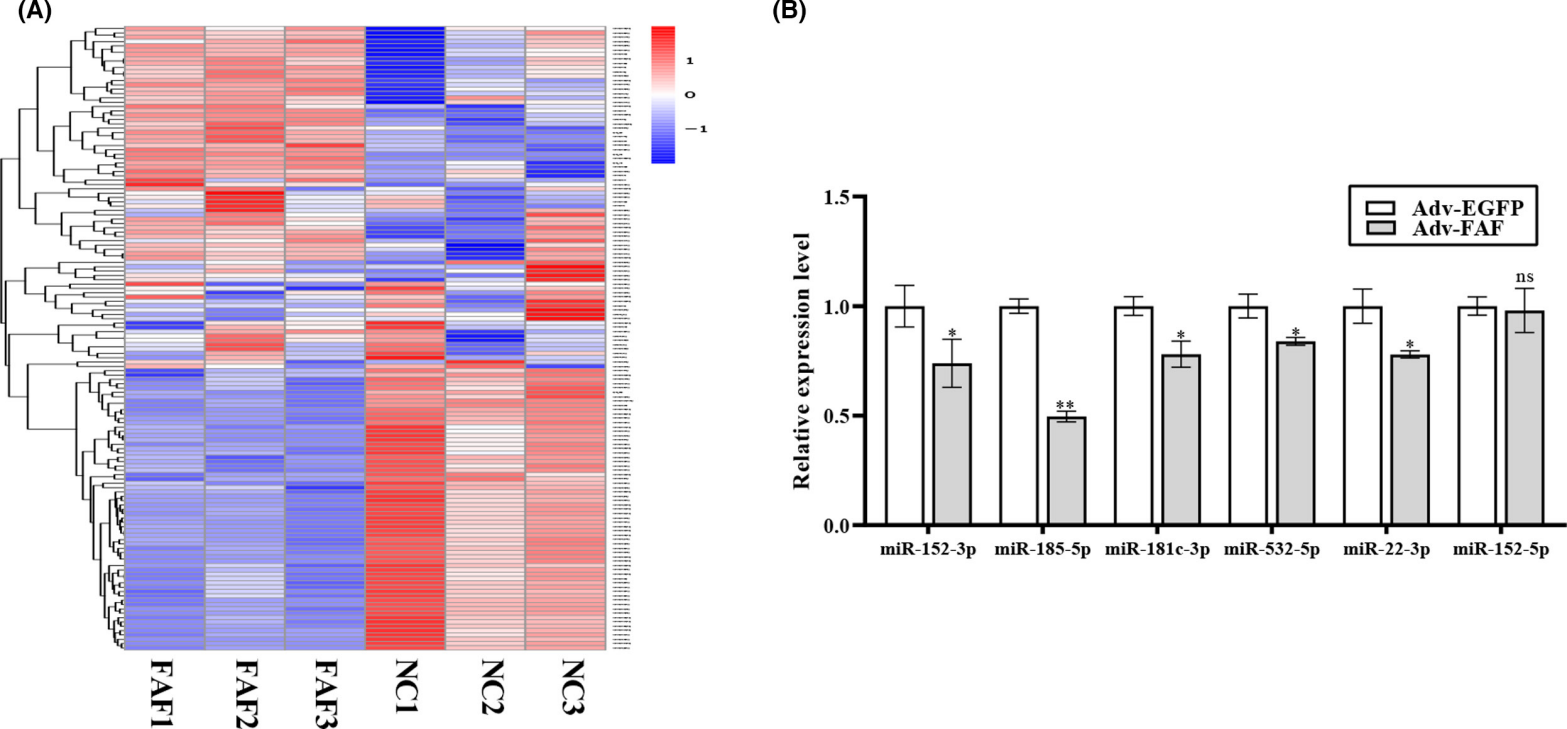
Expression profile of miRNAs in the FAF‐overexpressing NRCM and validation of potential target miRNAs. NRCM were transfected with Adv‐FAF and Adv‐EGFP, respectively. Subsequently, total RNA was extracted, and small RNA sequencing was performed to detect differentially expressed miRNAs in FAF‐overexpressing cardiomyocytes. (A) Heat map of the differentially expressed miRNAs between the control and FAF‐overexpressing groups. Relative expression levels are shown by the colour change. Higher expression is shown in red, whereas lower expression is shown in blue. (B) The expression levels of candidate miRNAs in Adv‐FAF‐transfected NRCM were determined by qRT‐PCR. *n* = 3, *p < 0.05, **p < 0.01, *vs* the Adv‐EGFP group

To further investigate how lncRNA FAF regulates pyroptosis under hypoxia, we performed small RNA sequencing to detect differentially expressed miRNAs in the FAF‐overexpressing cardiomyocytes. The differential expression profile is presented as a heat map (Figure 4A), and the screening criteria were absolute fold change >1.5, and adjusted *P*‐values <0.05. From the miRNA expression profile, there were a total of 140 differentially expressed miRNAs between the Adv‐FAF and Adv‐EGFP cardiomyocytes. Compared with the Adv‐EGFP group, 75 miRNAs had upregulated expression and 65 had downregulated expression. As previously described, lncRNA FAF is characterized as a cytoplasmic lncRNA,^22^ which may exert its regulatory function on pyroptosis by acting as a ceRNA for target miRNA.^25^ Therefore, in this particular scenario, we crosschecked the miRNAs downregulated in small RNA‐seq and CVDs that have been reported. On top of that, target miRNAs should contain putative binding sites for lncRNA FAF. A total of 6 candidate miRNAs met these criteria. qRT‐PCR was further conducted to verify the candidate miRNAs. Among all the candidates, miR‐185‐5p showed the most significant downregulation in Adv‐FAF cardiomyocytes (Figure 4B). Thus, miR‐185‐5p was selected for further analysis.

### 3.5 LncRNA FAF and PAK2 are direct targets of miR‐185‐5p in NRCM

**FIGURE 5:**
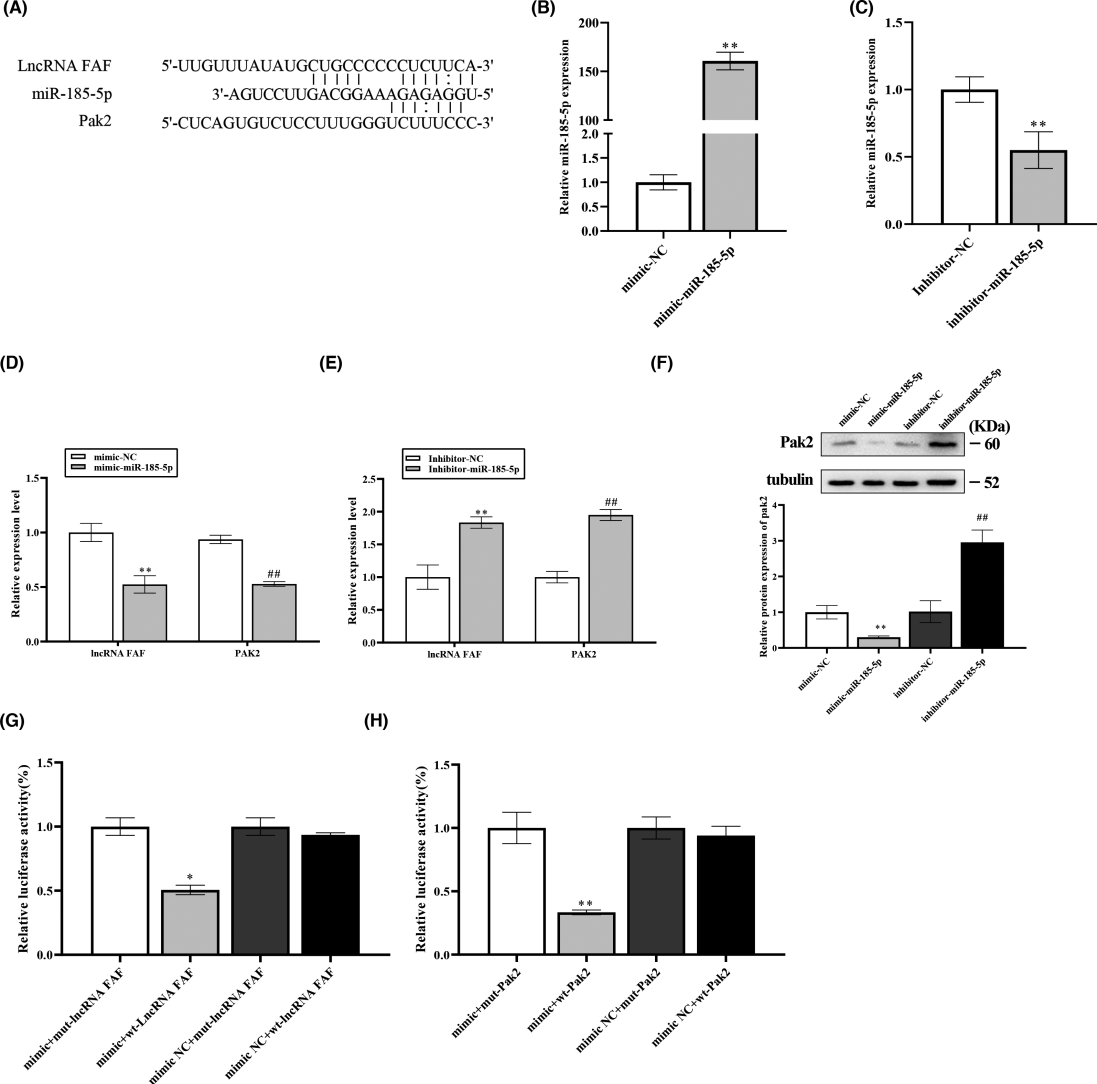
LncRNA FAF and PAK2 are direct targets of miR‐185‐5p in NRCM. (A) The binding sites of miR‐185‐5p with lncRNA FAF and PAK2 were predicted by RNAhybrid. (B and C) The transfection efficiency of miR‐185‐5p mimic and inhibitor in NRCM. (D–F) NRCM treated with miR‐185‐5p mimic or inhibitor were assessed for the expressions of lncRNA FAF and PAK2. *n* = 3, **p < 0.01 and ^##^ *p* < 0.01, *vs* the mimic NC group or inhibitor NC group. (G and H) Luciferase reporter assays were performed to verify the binding of miR‐185‐5p with lncRNA FAF and PAK2. *n* = 3, *p < 0.05, **p < 0.01, *vs* the mimic +mut‐lncRNA FAF or mimic +mut‐PAK2 group

RNAhybrid software was used to predict the potential target of miR‐185‐5p. Software analysis showed the putative target sites of miR‐185‐5p located in 3′‐UTR of PAK2 mRNA and lncRNA FAF (Figure 5A). To explore the regulatory effects of miR‐185‐5p on lncRNA FAF and PAK2, miR‐185‐5p mimic, NC‐mimic, miR‐185‐5p inhibitor or NC‐inhibitor were synthesized. The efficiency of miR‐185‐5p mimic and inhibitor transfection was confirmed by qRT‐PCR analysis (Figure 5B,C). Next, the expression of lncRNA FAF and PAK2 were measured in NRCM, which were transfected with miR‐185‐5p mimic, NC‐mimic, miR‐185‐5p inhibitor or NC‐inhibitor, respectively. After transfection of miR‐185‐5p mimics, lncRNA FAF and PAK2 levels were downregulated in cardiomyocytes. In contrast, lncRNA FAF and PAK2 levels were upregulated with the transfection of miR‐185‐5p inhibitor (Figure 5D‐F). These data indicated that miR‐185‐5p could target PAK2 and lncRNA FAF. To further examine the direct interaction among lncRNA FAF, miR‐185‐5p and PAK2, the putative and mutated miR‐185‐5p binding sequences from the PAK2 3’‐UTR and lncRNA FAF were cloned to luciferase reporter plasmids and luciferase activities was measured in HEK293 cells. As presented in Figure 5G and H, miR‐185‐5p mimic transfection significantly reduced luciferase activities in wt‐PAK2 and wt‐lncRNA FAF cells compared with NC‐mimic group, whereas transfection of miR‐185‐5p mimic did not change the luciferase activities in mut‐PAK2 and mut‐lncRNA FAF group. Overall, these findings revealed that FAF and PAK2 are direct targets of miR‐185‐5p in NRCM.

### 3.6 LncRNA FAF exerted antipyroptotic effects by targeting miR‐185‐5p/PAK2

**FIGURE 6:**
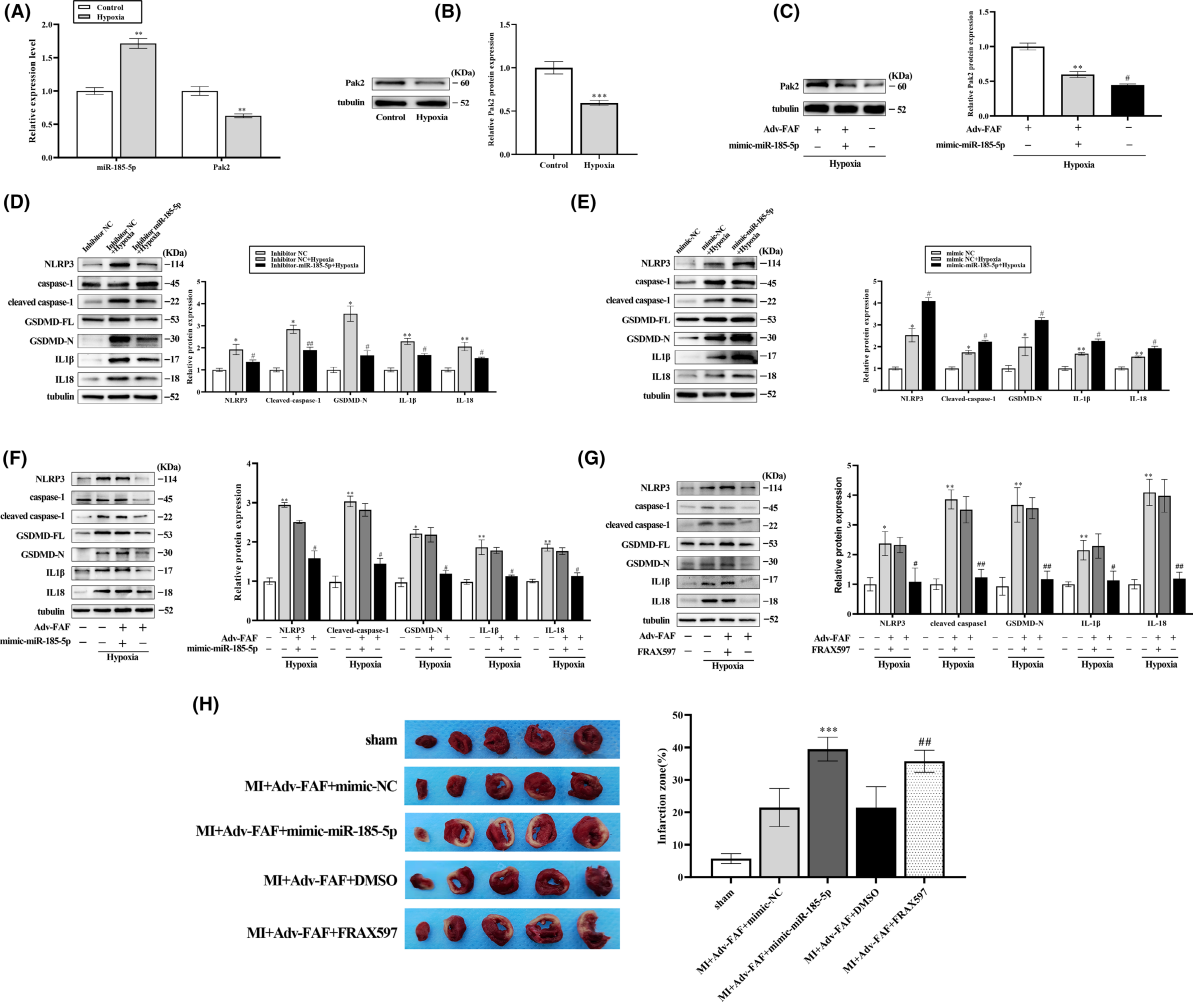
LncRNA FAF exerted antipyroptotic effects by targeting miR‐185‐5p/PAK2. (A and B) The differential expression of miR‐185‐5p and PAK2 between hypoxic and normoxic cardiomyocytes. (C) The protein expression of PAK2 in hypoxic NRCM transfected with Adv‐FAF and/or miR‐185‐5p mimics was determined by Western blots. n=3, **p < 0.01, *vs* the control group, # *vs* the Adv‐FAF+mimic‐miR‐185‐5p+hypoxia group. (D‐E) Transfection of miR‐185‐5p inhibitor contributed to downregulating the pyroptosis‐related protein expression, whereas miR‐185‐5p mimic transfection upregulated pyroptosis‐related protein expression. n=3, *p < 0.05, **p < 0.01, *vs* the inhibitor NC or mimic NC group, #*p* < 0.05, ##*p* < 0.01 *vs* the inhibitor NC+hypoxia group or mimic NC+hypoxia group. (F‐G) Transfection of miR‐185‐5p mimics or FRAX597 abolished the antipyroptotic effects of lncRNA FAF overexpression in hypoxic NRCM. *n* = 3, *P*p < 0.05, **P**p < 0.01, *vs* the control group, *^#^p* < 0.05, *^##^p* < 0.01 *vs* the Adv‐FAF +mimic‐miR‐185‐5p + hypoxia group or Adv‐FAF +FRAX597 + hypoxia group. (H) The infarct size of rats after regulating miR‐185‐5p/PAK2 axis was measured by TTC staining. *n* = 5 rats per group, ^***^p < 0.001, *vs* the MI +Adv‐FAF +mimic‐NC group; *^##^p* < 0.01 *vs* the MI +Adv‐FAF +FRAX597 group

It has been reported that regulation of miR‐185‐5p and PAK2 could exert cardioprotective effects. On that basis, we further examined the functional relationship between lncRNA FAF and miR‐185‐5p/PAK2 in hypoxia/ischmia‐treated NRCM and ischaemic heart. In vitro, upregulation of miR‐185‐5p expression was detected in the hypoxia/ischaemia injury group, while downregulation of PAK2 expression was found in cardiomyocytes exposed to hypoxia and ischaemia (Figure 6A,B). Overexpressing lncRNA FAF upregulated PAK2 protein levels under hypoxic conditions, which was reversed by transfection of miR‐185‐5p mimics (Figure 6C). In addition, we examined the relationship between lncRNA FAF and PAK1, a close member of PAK2, under hypoxia. The data showed that the expression level of PAK1 was decreased in NRCM subjected to hypoxia/ischaemia condition, while overexpression of lncRNA FAF had no effect on PAK1 in NRCM (Figure S2). The expression of pyroptosis‐related proteins was evaluated by Western blot. As shown in Figure 6D,E, transfection of the miR‐185‐5p inhibitor contributed to the mitigation of cell pyroptosis. In contrast, miR‐185‐5p mimic aggravated cell pyroptosis under hypoxia‐ischaemia. More importantly, overexpressing‐lncRNA FAF inhibited pyroptosis in NRCM, while these effects of lncRNA FAF were abolished by cotransfection of miR‐185‐5p mimic or FRAX597 (Figure 6F,G). It should be noted that both miR‐185‐5p inhibitor and PAK2 expression plasmid fail to further enhance the antipyroptotic effects in FAF‐overexpressed NRCM (Figure S4). These data suggested that both miR‐185‐5p and PAK2 act as the downstream target of lncRAN FAF in regulating pyroptosis in NRCM. Similarly, TTC staining analysis showed that overexpression of miR‐185‐5p or inhibition of PAK2 activity could increase the infarct size and attenuate the protection of lncRNA FAF in vivo (Figure 6H). Thus, combined with all the above findings, lcnRNA FAF/miR‐185‐5p/PAK2 axis plays a vital role in regulating myocardial pyroptosis both in vivo and in vitro.

## 4 DISCUSSION

Pyroptosis is considered as inflammasome‐activated programmed cell death in response to various pathogens and host danger signals.^39^ Emerging studies have revealed that pyroptosis plays an important role in the pathological process of MI and understanding its regulatory mechanism is urgently needed for developing new treatment strategies for MI.^40^ Previously, Li et al.^41^ found that overexpression of GDF11 repressed cell pyroptosis and alleviated MI injury. Zhang et al.^42^ emphasized the cardioprotective effects of metformin against myocardial ischaemia/reperfusion (I/R) injury based on the pyroptosis mechanism. Mao et al. suggested that lncRNA KLF3‐AS1 derived from exosomes could ameliorate pyroptosis of cardiomyocytes and attenuate MI progression. In prior studies, lncRNA FAF was demonstrated to be involved in MI and could regulate the apoptosis of cardiomyocytes and fibrosis in cardiac fibroblasts by altering the transcription of FGF9.^22^, ^23^ In the present study, we firstly demonstrated that overexpression of lncRNA FAF with adenovirus exhibited antipyrotpotic effects both in vivo and in vitro. Improved cardiac function and reduced infarct size were also observed in FAF‐overexpressed MI rats. In addition, detection of pyroptosis‐related proteins in rat MI models and hypoxia NRCM supported that overexpressing lncRNA FAF can repress the expression levels of NLRP3, caspase‐1, GSDMD, IL‐1β and IL‐18. Of note, the activation of NLRP3 inflammasome was significantly inhibited with the overexpression of lncRNA FAF in hypoxia/ischaemia injured cardiomyocytes. Meanwhile, interfering with lncRNA FAF promoted pyroptotic cell death and the assembly of NLRP3 inflammasome in NRCM under hypoxia/ischaemia. These findings collectively suggested that lncRNA FAF may regulate pyroptosis by directly or indirectly targeting NLRP3 inflammasome during MI.

LncRNAs exert biological function through various mechanisms, including chromatin organization, epigenetic modification, transcriptional and translation regulation.^19^, ^43^ In our previous study, lncRNA FAF exerts its biological function by regulating gene transcription in nucleus.^22^, ^23^ It is worth noting that lncRNAFAF is characterized as a cytoplasmic lncRNA, which tends to modulate gene expression by acting as a miRNA sponge in cytoplasm. In this study, we further investigated the potential molecular mechanism of lncRNA FAF acting as a miRNA sponge. We utilized small RNA sequencing and luciferase reporter assays to determine the candidate miRNAs. MiR‐185‐5p expression was found to be significantly downregulated in small RNA profiles, which was further validated by qRT‐PCR. Luciferase reporter assays were applied to confirm that lncRNA FAF could sponge miR‐185‐5p and further upregulate PAK2 expression. Collectively, we demonstrated that miR‐185‐p/PAK2 is a novel target of lncRNA FAF in NRCM.

MiR‐185‐5p has been reported to participate in the process of cell death in response to various cell stresses, including hypoxia/ischaemia injury.^27^, ^28^, ^44^ A recent study indicated that inhibition of miR‐185‐5p in endothelial cells contributed to the recovery of heart function after MI.^29^ However, the exact mechanism by which miR‐185‐5p regulates cardiomyocyte pyroptosis after hypoxia‐ischaemia remains unknown. In this study, our data firstly exploited that NRCM with downregulation of miR‐185‐5p expression resisted pyroptosis‐induced injury and upregulated the expression of lncRNA FAF and PAK2. In contrast, upregulation of miR‐185‐5p expression in NRCM exacerbated cell pyroptosis and reduced the expression of lncRNA FAF. Moreover, forced overexpression of miR‐185‐5p attenuated the protection of lncRNA FAF both in hypoxia/ischaemia‐treated NRCM and in ischaemia myocardium.

Accumulating evidence suggests that excessive endoplasmic reticulum (ER) stress can activate the NLRP3 inflammasome through multiple mechanisms, such as oxidative stress, Ca^2+^ release and NF‐κB activation.^45^ PAK2, P21 activated kinase 2, is a member of the group I PAK family and plays a pivotal role in maintaining ER homeostasis under stress conditions. PAK2 is also considered cardioprotective in several cardiovascular diseases.^46^, ^47^ In the mice I/R model, the upregulation of PAK2 repressed ER stress and inhibited cardiomyocyte apoptosis.^48^ In this study, we elaborated for the first time the potential roles of PAK2 in regulating cell pyroptosis. Our findings suggested that PAK2 is downregulated in hypoxia cardiomyocytes and lncRNA FAF could modulate PAK2 expression by acting as a ceRNA to bind with miR‐185‐5p. More importantly, inhibiting PAK2 activity by FRAX597 abolished the antipyroptotic effects of lncRNA FAF both in vivo and in vitro. Hence, it is reasonable to speculate that lncRNA FAF can reduce ER stress by miR‐185‐5p/PAK2 axis and further exert its antipyroptotic effect through inhibition of NLRP3 inflammasome activation in MI. Interestingly, PAK2 has been reported to inhibitor cell apoptosis via targeting caspase‐7.^49^ In the present study, we also found that lncRNA FAF could regulate caspase‐7 activity under hypoxia/ischaemia in NRCM (Figure S3). However, more efforts are needed to understand its mechanism.

In summary, our study investigated the role of lncRNA FAF and its mechanism on cardiomyocyte pyroptosis. In both in vivo and in vitro experiments, we demonstrated that lncRNA FAF could alleviate cardiomyocyte pyroptosis, enhance cell viability and improve cardiac function through sponging miR‐185‐5p and thus to promote PAK2 expression. Our work may shed new light on the therapeutic potential of lncRNA FAF for future AMI treatment.

## CONFLICTS OF INTEREST

There are no conflicts of interest.

## AUTHOR CONTRIBUTION

**Jie Gu:** Conceptualization (equal); Data curation (equal); Methodology (equal); Validation (lead). **Jian‐Zhou Shi:** Formal analysis (equal); Investigation (equal); Project administration (supporting); Validation (equal); Visualization (lead). **Ya‐xing Wang:** Formal analysis (equal); Methodology (supporting); Visualization (equal); Writing – original draft (equal). **Liu Liu:** Formal analysis (supporting); Methodology (supporting); Validation (equal); Writing – original draft (supporting). **Si‐Bo Wang:** Data curation (supporting); Investigation (supporting); Methodology (supporting); Software (supporting); Validation (supporting); Writing – original draft (supporting). **Jia‐Teng Sun:** Project administration (supporting); Resources (supporting); Validation (supporting); Writing – original draft (supporting). **Tian‐Kai Shan:** Data curation (supporting); Methodology (supporting); Visualization (supporting). **Hao Wang:** Conceptualization (supporting); Project administration (supporting); Validation (supporting); Writing – review & editing (supporting). **Qi‐Ming Wang:** Conceptualization (lead); Project administration (equal); Resources (equal); Supervision (equal). **Lian‐Sheng Wang:** Project administration (lead); Resources (lead); Supervision (equal).

## Supporting information

Supplementary Material

## Data Availability

The data that support the findings of this study are available from the corresponding author upon reasonable request.
