# Supplementary material for: LncRNA FAF attenuates hypoxia/ischaemia‐induced pyroptosis via the miR‐185‐5p/PAK2 axis in cardiomyocytes
Source: J Cell Mol Med. 2022 Apr 3;26(10):2895–907. doi: 10.1111/jcmm.17304 (PMC9097851; doi:10.1111/jcmm.17304)
Supplement: Supplementary file 1 — Supplementary Material [file JCMM-26-2895-s001.docx]

Table S1

Specific primer list

| Target primers | Sequences |
| --- | --- |
| U6-F | CTCGCTTCGGCAGCACA |
| U6-R | AACGCTTCACGAATTTGCGT |
| miR-152-3p-F | CGCGTCAGTGCATGACAGA |
| miR-152-3p-R | AGTGCAGGGTCCGAGGTATT |
| miR-152-5p-F | GCGCGAGGTTCTGTGATACACT |
| miR-152-5p-R | AGTGCAGGGTCCGAGGTATT |
| miR-181c-3p-F | GCGACCATCGACCGTTGAG |
| miR-181c-3p-R | AGTGCAGGGTCCGAGGTATT |
| miR-532-5p-F | CGCGCATGCCTTGAGTGTAG |
| miR-532-5p-R | AGTGCAGGGTCCGAGGTATT |
| miR-22-3p-F | GCGAAGCTGCCAGTTGAAG |
| miR-22-3p-R | AGTGCAGGGTCCGAGGTATT |
| PAK1-F | GCTTCTCCCATTTCCTGAT |
| PAK1-R | CCATGCCCACAAGCTAA |
| 18S-F | TCAAGAACGAAAGTCGGAGG |
| 18S-R | GGACATCTAAGGGCATCAC |

*F* forward, *R* reverse


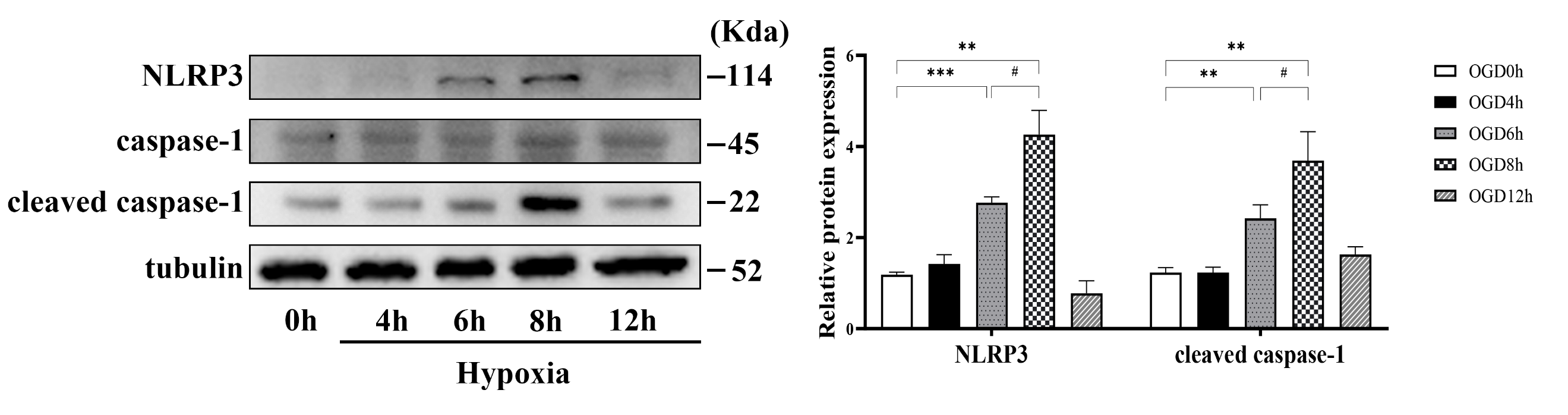
**Supplemental Figure S1.** **Western blot analysis of time-dependent effects on pyroptosis-related proteins after hypoxia.** NRCM were exposed to hypoxia-ischemia condition for 0 hour, 4 hours, 6 hours, 8 hours, 12 hours. NLRP3, caspase-1 and cleaved caspase-1 were determined by western blot. *n* = 3, **P* < 0.05, ***P* < 0.01, ****P* < 0.001 *vs* the 0h group, *^#^P* < 0.05 *vs* the 8h group.


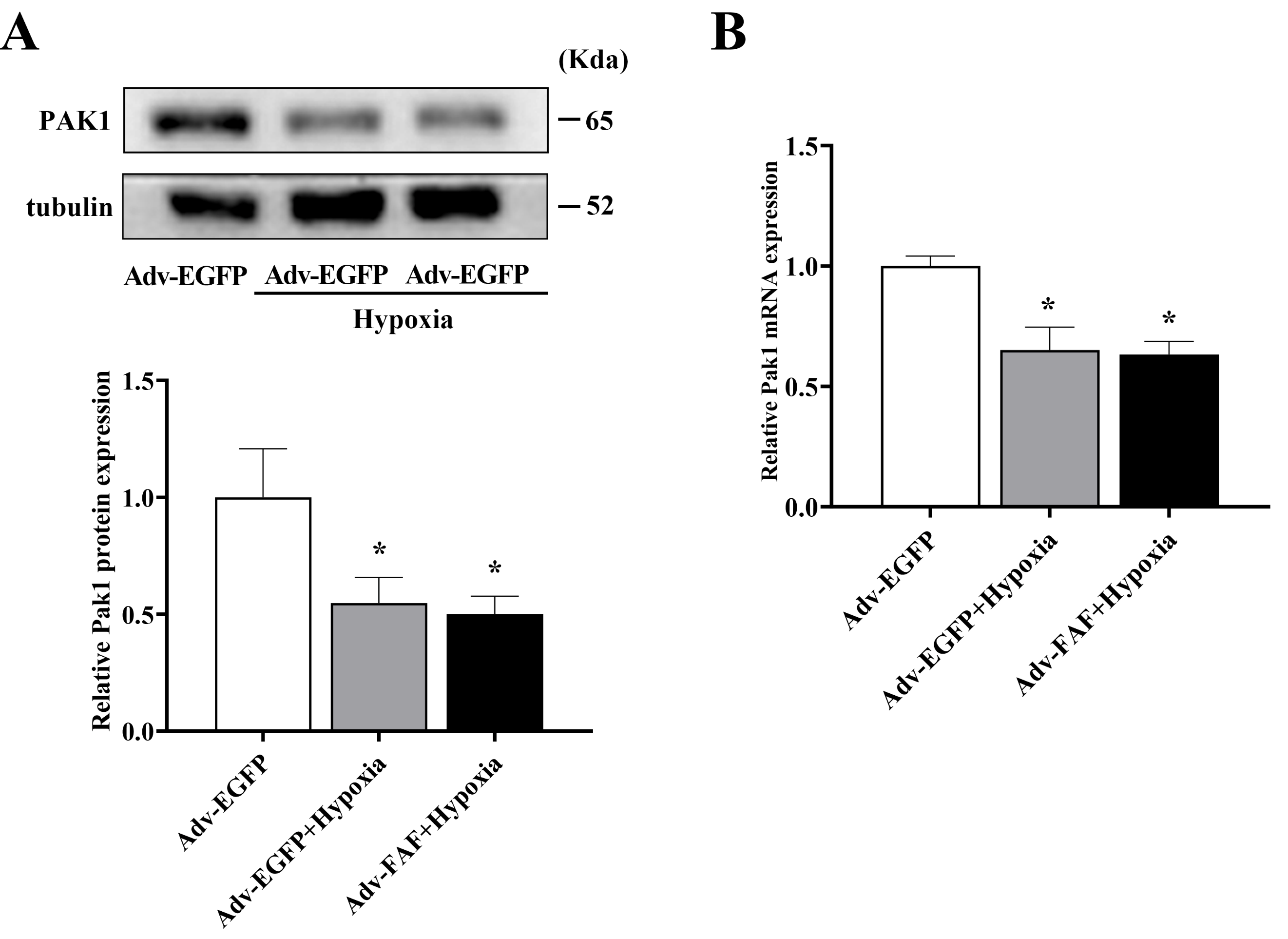


**Supplemental Figure S2. The expression of Pak1 in FAF-overexpressed NRCM under hypoxia-ischemia.**

NRCM were transfected with Adv-lncRNA FAF or Adv-EGFP for 12 hours following hypoxia. (A) The protein level of PAK1 was determined by western blot. (B) The mRNA level of PAK1 was determined by qRT-RCR. *n* = 3, **P* < 0.05 vs the Adv-EGFP group.


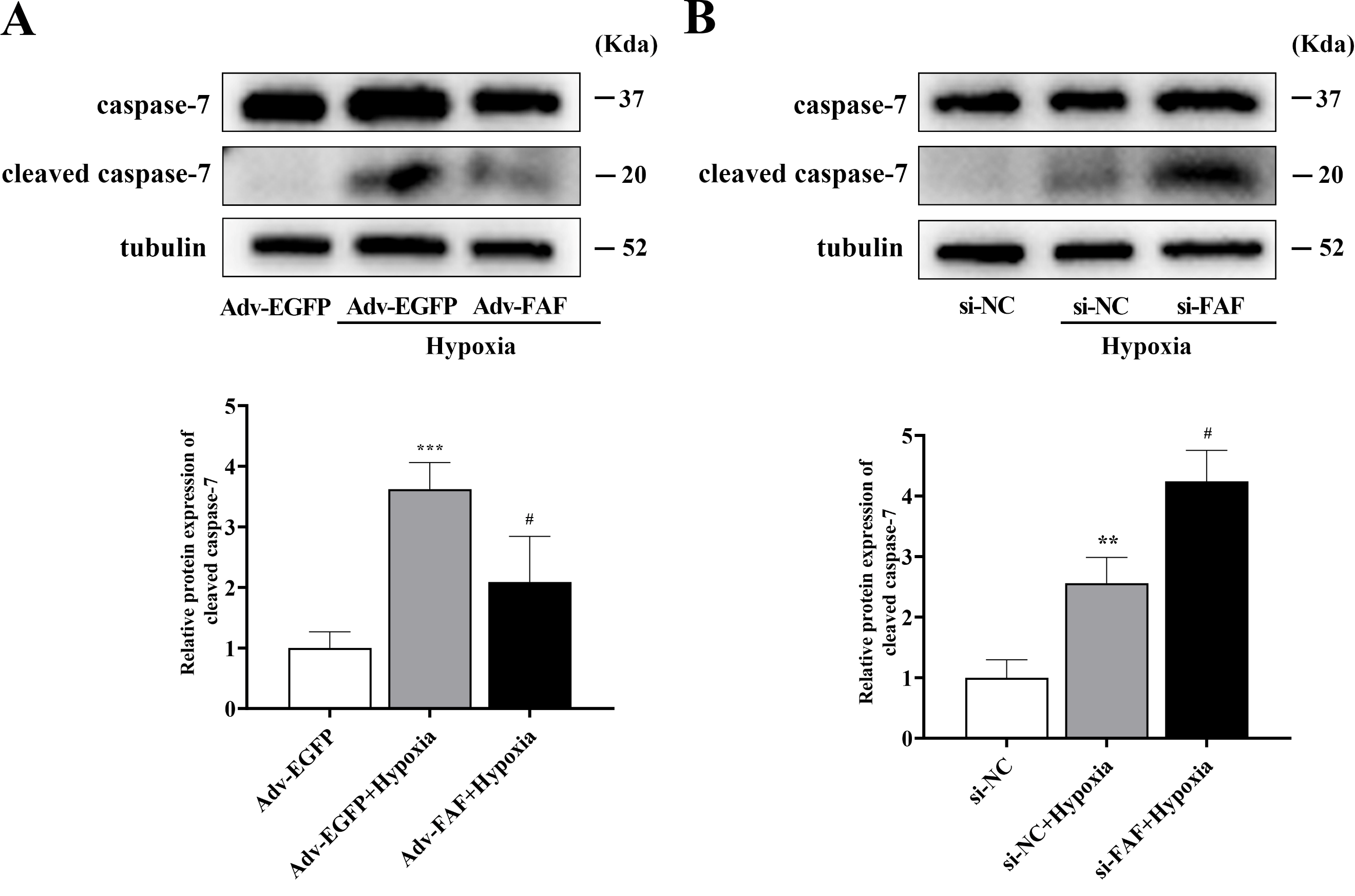


**Supplemental Figure S3. LncRNA FAF regulates the cleavage of caspase-7 in NRCM under hypoxia.** NRCM were transfected with Adv-FAF or Si-FAF for 12 hours following hypoxia. Caspase-7, cleaved caspase-7 were determined by western blot. *n* = 3, ***P* < 0.01, ****P* < 0.001 vs the Adv-EGFP or the si-NC group, *^#^P* < 0.05 *vs* the Adv-EGFP + Hypoxia or the si-NC + Hypoxia group.


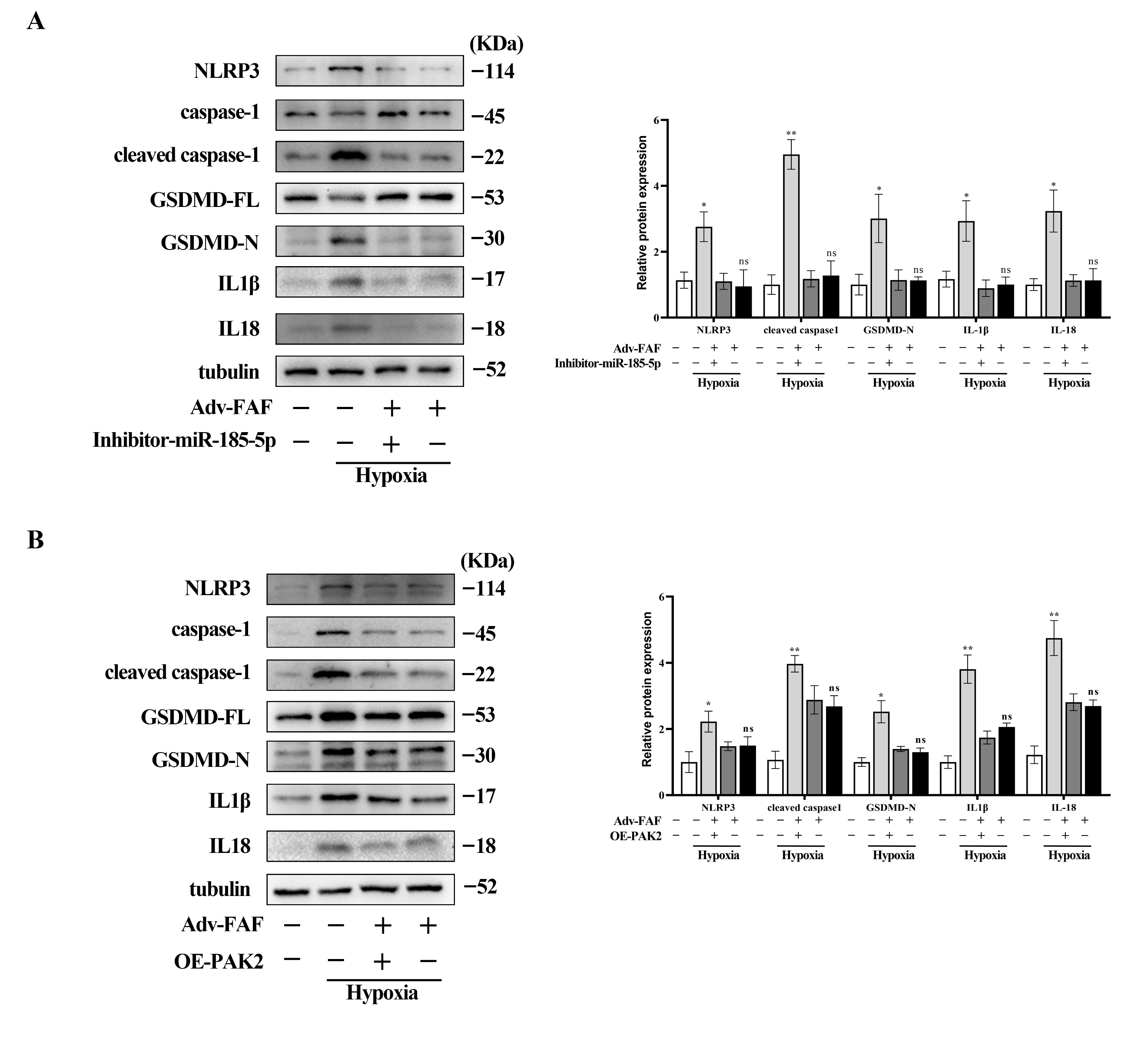


**Supplemental Figure S4. The expression of pyroptosis-related proteins in FAF-overexpressed NRCM cotransfected with miR-185-5p inhibitor or PAK2 expression plasmid under hypoxia-ischemia.** (A) NRCM were cotransfected with Adv-FAF and miR-185-5p mimic for 12 hours following hypoxia. Pyroptosis-related proteins were determined by western blot. (B) NRCM were cotransfected with Adv-FAF and PAK2 expression plasmid for 12 hours following hypoxia. Pyroptosis-related proteins were determined by western blot. *n* = 3, **P* < 0.01, ***P* < 0.001 vs the control group, ns means *P* value > 0.05 *vs* the Adv-EGFP + inhibitor-miR-185-5p + hypoxia or the Adv-EGFP + OE-PAK2 + hypoxia group.
